# Examining how different social account timings influence stress resolution in the aftermath of a psychological contract breach

**DOI:** 10.1038/s41598-022-25728-8

**Published:** 2022-12-20

**Authors:** Safâa Achnak, Thomas Rigotti, Tim Vantilborgh

**Affiliations:** 1 Work and Organizational Psychology (WOPs), Vrije Universiteit Brussel, Brussels, Belgium; 2 Arbeits-, Organisations- und Wirtschaftspsychologie, Johannes Gutenberg-University Mainz, Mainz, Germany

**Keywords:** Human behaviour, Physiology, Psychology

## Abstract

A great deal of empirical research on the consequences of a psychological contract breach (PCB) has overlooked the role of time in understanding individuals’ reactions to a PCB. Moreover, psychological contract research primarily focuses on how employees react to perceptions of a PCB, while questions regarding how the organization’s responsiveness (i.e., social account) might impact these reactions remain unanswered. We aimed to enhance the understanding of stress reactions and recovery that are triggered by PCB perceptions and stimulate empirical research that treats psychological contracts as a dynamic phenomenon. Drawing on the conservation of resources theory, we investigated how social account delivery timing—and its subjective experience—influences individuals’ stress resolution processes in the aftermath of a PCB. To this end, we used an experimental design and assessed participants’ physiological (i.e., heart rate) and psychological (i.e., self-report) stress reactions after inducing a breach. Our results underscore that a PCB is experienced as a stressful event. In addition, we find that social account timing influences heart rate recovery following a PCB. We discuss the theoretical and practical implications of our findings and offer recommendations for practitioners.

## Introduction

Early theorizing on employee−employer exchanges (e.g.,^1,2^) highlighted the importance of the psychological contract (PC) to understanding the employment relationship. PC is defined as a cognitive schema containing an employee’s perceptions of the mutual obligations between the employee and an employer^3^. To date, considerable attention has been paid to the negative consequences of employees’ perceptions of a psychological contract breach (PCB; i.e., a perceived negative discrepancy between employer obligations and delivered inducements^4–6^). For instance, a PCB was associated with feelings of violation (i.e., a mixture of negative emotions, such as anger and frustration^7^), which is known to negatively impact various work-related outcomes, such as organizational commitment, performance, and job satisfaction^8,9^. In addition, feelings of violation harm employees’ well-being by provoking stress, strain, anxiety, depression, and burnout^10–14^. A great deal of empirical research on PCs is aimed primarily at understanding how employees react to perceptions of a PCB, while remarkably little empirical research is devoted to organizational responses after breaches have occurred (e.g.,^15–17^).

Little attention has also been given to the role of time in understanding reactions to a PCB^3,18^. This is surprising since it is generally accepted that the PC concept is inherently dynamic (e.g.,^19,20^). PCs can be breached or violated, maintained, repaired, abandoned, or renegotiated throughout the employment relationship^3,20^. Accounting for this dynamism is necessary to discern how reactions to a PCB may aggravate or diminish over time and which conditions initiate and influence such changes^18^. We addressed this temporal issue by experimentally studying stress reactions to perceptions of a PCB as a *process* (i.e., how stress responses fluctuate, evolve, and develop over time following a breach). Ployhart and Vandenberg^21^ defined this research as *descriptive time-based*, as the aim is to describe how the trajectory of a focal variable (i.e., stress response) changes over time. We go a step further by modeling time as an explanatory factor for different trajectories after PCBs. We argue that the *timing* in which organizational repair efforts are made, and thus resources are made available, can impact the success (or lack thereof) of the stress recovery *process* following initial stress reactions after a PCB, as proposed by Tomprou et al.^22^ in their post-violation model. We draw upon the conservation of resources theory (COR theory^23^) to explain why the timing of social accounts matters in this stress recovery process. Social accounts can be described as attempts by employers to shape employee perceptions following a negative event^24^. Employers use this process to bridge the gap between what employees initially expected and what they currently perceive^25^.

Time is commonly studied as an objective construct, measured in seconds, minutes, hours, or years. However, the way individuals think about and perceive time and duration within their workplace is an important source of information for understanding individuals’ responses to work events. We, therefore, follow Shipp and Cole’s^26^ recommendation to incorporate a complete temporal view with both objective and subjective time measures. More specifically, the present research distinguishes between the objective duration of the resolution process (standard clock) and the perceived duration of the resolution process, referred to as *resolution velocity* in Tomprou and colleagues’^22^ post-violation model. Time perception literature suggests that the objective duration in which events occur can be distorted by individual and situational factors^27,28^. When studying the timeliness of social accounts and their effect on stress resolution, it becomes relevant to differentiate objective from subjective time durations.

To gain a broad understanding of stress responses following perceptions of a PCB, we used a combination of both physiological and self-reported measures. The majority of PC studies examining emotional and stress reactions to a PCB employed retrospective self-reported measures. However, research has shown that memories of emotions are prone to systematic biases, such as current feelings about appraisals of past events^29^. Moreover, physiological measures make it possible to study information that exists outside of individuals’ conscious awareness^30^. Accordingly, using physiological measures leads to more objective and unprejudiced evaluations of stress responses to a PCB. We, therefore, assessed participants’ physiological reactions through their heart rate (HR) in addition to psychological stress responses.

We contribute to research on the effect of responses to a PCB in several ways. By using an experimental between-person design and manipulating the timing of social accounts after a PCB, we provide evidence for a causal link between timing and the trajectory of the stress response (cf.,^31^). By measuring both time and the stress response subjectively as well as objectively, we can comprehensively test our theoretical claim that responding to a PCB early will result in better outcomes. Moreover, by integrating COR theory into PC theory, we provide further insights into how organizational resources play a role in different post-violation outcomes.

### Stress reactions to psychological contract breach

COR theory states that individuals have a need and desire to retain, protect, and build valuable resources, such as time, money, health, and relationships^23^. When individuals perceive a loss of resources, a threat of losing resources, or a lack of resource gain following the investment of resources, they experience stress^23,32^. Evidence suggests that the threat of resource loss triggers anticipatory stress, meaning that the belief that one might lose resources can be equally detrimental as actually losing resources^33^. From a COR perspective, the PC contains an employee’s beliefs regarding the exchange of resources between two parties (e.g., career development opportunities in exchange for loyalty). Following this, a PCB creates a perceived imbalance between investments and outcomes, which triggers a resource loss process. This loss process will, in turn, elicit stress reactions^34^. In contrast, when the organization fulfills its perceived obligations (i.e., PC fulfillment), desired resources can be maintained and/or acquired, which will prevent employees from experiencing such reactions^35^. When employees monitor their PC for discrepancies and perceive PC fulfillment, they are expected to experience low arousal emotions^3^. Hence, we expect no changes in experienced stress when the PC is fulfilled and hypothesize the following:

#### Hypothesis 1a

Perceptions of a PCB cause increased psychological stress responses compared to perceptions of PC fulfillment.

In addition to a psychological stress response, the threat of losing resources can also give rise to physiological changes. It has been argued that these physiological responses act as a mechanism through which the negative effects of the threat or actual loss of resources can be exacerbated^36^. That is, threat appraisals are associated with constrictive physiological stress responses characterized by the activation of the sympathetic-adrenal-medullary system. The activation of this system provokes a release of hormones (i.e., adrenaline and noradrenaline) that, in turn, increase HR^37,38^. We, therefore, argue that individuals facing a PCB will experience increased physiological reactivity in the form of increased HR in addition to increased psychological stress.

#### Hypothesis 1b

Perceptions of a PCB cause increased physiological reactivity compared to perceptions of PC fulfillment.

### The use of social accounts as a social resource in the aftermath of a PCB

From the COR perspective, when losses occur, individuals are motivated to apply resource conservation strategies, during which they employ available individual (e.g., personal) or contextual (e.g., social accounts) resources to cope successfully^39^. In the case of successful coping, new resources are generated, which enables resource replenishment and neutralizes the conditions that activated resource losses. In contrast, unsuccessful adaptation to stress leads to resource diminishment, which results in negative functional and emotional outcomes^35^. Social accounts, considered a form of interactional justice, may be an important social resource facilitating successful coping, as they can address justice issues that emerge when workplace events occur. More specifically, when employees perceive interactional justice, such as when their employer invests time in explaining the reasons behind job-related decisions and provides legitimate justifications for workplace events, it results in greater respect and consideration toward their employers^25,40^. These perceptions may signal that employees hold a dignified position within the organization, which promotes a positive sense of self and fuels employees’ positive energy to overcome challenges^17,41^. These feelings of self-worth and self-mastery may serve as important personal resources, as they promote the capability of withstanding stress^42^. As resources tend to generate other resources, we argue that providing employees with a social account serves as an important social resource that positively affects the stress resolution process.

### The role of time in social account delivery

While we have a wealth of knowledge on the effectiveness of different social account types (e.g., apology, justification, denial, offering compensation^24,43,44^), little knowledge exists about the role the timeliness of such accounts play in the resolution process (e.g.,^45^). Knowledge about the temporal aspect of social accounts is crucial since it can help us understand why some organizational interventions after a PCB are rejected while others are accepted. Hence, it allows managers and practitioners to predict more accurately the effectiveness of the social accounts they provide. We, therefore, aimed to address this gap by experimentally examining how different social account delivery timings (i.e., prompt, delayed, late) affect the unfolding stress reactions following perceptions of a PCB. Existing empirical research on the timing of social accounts has often referred to whether the account was provided before or after a discrepant event (e.g.,^46^). However, since organizations are not always able to anticipate perceptions of a PCB, we focus here on retrospective social accounts, that is, social accounts given *after* a PCB has been perceived.

Over the past decade, scholars have been increasingly studying how the dynamics of resources in COR theory depend on time (e.g.,^47–49^). For example, based on the different principles and corollaries of COR theory, Halbesleben and colleagues^50^ outlined a series of potential resource trajectories one might experience within a well-defined episode. Several of these trajectories (i.e., trajectories 2 [downward resource fluctuation], 4 [resource loss cycle], and 5 [resource passageway]) match possible PCB situations where the investment of resources does not generate desired or expected returns (after initial investment—and even potential gain—of resources, there is a sudden and ongoing loss of resources). Once resources reach lower levels, employees are less equipped to address ensuing stressors, which can provide an additional source of strain, culminating in a loss spiral. As proposed by Hobfoll and colleagues^51^, the role that time plays in such dynamics can be studied by examining the specific timing at which a resource becomes available relative to the timing of resource loss. This corresponds to what we aimed to examine in this research.

More specifically, we aimed to study how different timings in which the social account as a social resource is offered influence the post-breach stress recovery process. Hence, the timing of social accounts is treated as a focal construct, as we focus explicitly on the meaning of time in the dynamic stress resolution process (cf.^26^). We start by examining the timing of social accounts as a function of objective time (i.e., clock time). However, to better understand the timing of social accounts, we must consider the time scale on which the stress resolution process occurs. No clear guidelines currently exist in the PC literature on the ideal time scale of PCB effects. This issue is further complicated by the fact that this time scale may differ between types of obligations being breached and may depend on individual and contextual factors^52^. In particular, the time scale for social accounts should be considered from the perspective of how the individual brackets the experience into a meaningful episode^53^. For example, in the context of an experiment such as ours, individuals likely view the duration of the experiment itself as a meaningful episode. Put differently, a social account that is offered after 30 min might be considered late in a 1-h experiment, whereas it might be considered a swift response in an organizational context. Prompt, delayed, and late timing of social accounts should, therefore, be interpreted within the time scale of a 1-h experiment in the present study (In the context of this experiment, prompt indicates that a social account is offered immediately following the PCB, delayed means that a social account is offered one block after the PCB is induced (midway between PCB and the end of experiment), and delayed means that a social account is offered two blocks after the PCB is induced (at the end of the experiment)). We expect that prompt timing will be positively associated with more successful stress trajectories, preventing them from further depleting personal resources to deal with the emotional demands of the stressor. By postponing social account timing, employees are more likely to experience trajectories in which stress only decreases partially, as they need to cope with the resource loss for a longer amount of time (i.e., delayed and late timing conditions). In the worst case, employees may enter an escalating spiral of losses, where each loss may lead to further depletion of resources needed for the next threat of loss (i.e., no social account condition). We, therefore, propose that the timeliness of a social account will influence stress resolution linearly and negatively.

#### Hypothesis 2a

A negative relationship exists between social account timeliness and psychological stress recovery; meaning, the longer it takes for a social account to be delivered, the less likely it is that an individual’s psychological stress level will return to pre-breach levels.

#### Hypothesis 2b

A negative relationship exists between social account timeliness and physiological stress recovery; meaning, the longer it takes for a social account to be delivered, the less likely it is that an individual’s physiological stress level will return to pre-breach levels.

### The subjective interpretation of time: resolution velocity

In the previous section, we hypothesized that the objective duration between a PCB and social account delivery will impact individuals’ stress resolution processes. In the present section, we argue that not only will the objective duration influence the stress resolution process but also how individuals subjectively perceive this duration^54^. Indeed, many scholars have suggested that individuals experience time both objectively and subjectively (e.g.,^55–57^). Drawing on the time perception literature, we argue that perceived duration can be distorted by different factors, such as attention shifts^27^, visual onsets^58^, emotions^59^, and mood disorders^28^. Since objective and perceived timing can differ, it becomes crucial to distinguish between the two concepts when examining the temporal aspect of social accounts. To understand how the perceived subjective timing of social accounts influences stress recovery following a PCB, we turn to the concept of resolution velocity from self-regulation theory^60^, which has already been proposed to matter in the context of PCs^22^.

Self-regulation theory proposes that a PCB triggers a discrepancy-reducing feedback loop, as individuals try to reduce the discrepancy between obligated and delivered inducements^22,60^. This feedback process is accompanied by a second loop that focuses on the perceived velocity of discrepancy reduction^60^. When the rate of change in the discrepancy reduction is slower than desired, negative affect will be experienced, while positive affect is experienced when the rate of change in the discrepancy reduction is faster than desired^60^. As a social account forms a social resource that may help to reduce the discrepancy, the perceived speed or velocity with which a social account is offered will thus affect the emotional response. This aligns with COR theory, which suggests that the outcome of this self-regulation process will be important in determining whether an individual experiences a loss cycle^50^.

To summarize, we propose that individuals will compare the perceived velocity of resolution efforts (i.e., the perceived time elapsed between a PCB and social account delivery) with their own desired speed or velocity^61^. If individuals consider the duration between a PCB and the ensuing social account to be slower than the desired velocity (i.e., low-resolution velocity), they are more likely to experience negative affect and stress, whereas positive affect is generated and stress is reduced when the duration is perceived to be faster than the acceptable velocity (i.e., high-resolution velocity^60^). We, therefore, hypothesize:

#### Hypothesis 3a

A positive relationship exists between resolution velocity and psychological stress recovery.

#### Hypothesis 3b

A positive relationship exists between resolution velocity and physiological stress recovery.

## Method

### Procedure

**Figure 1:**
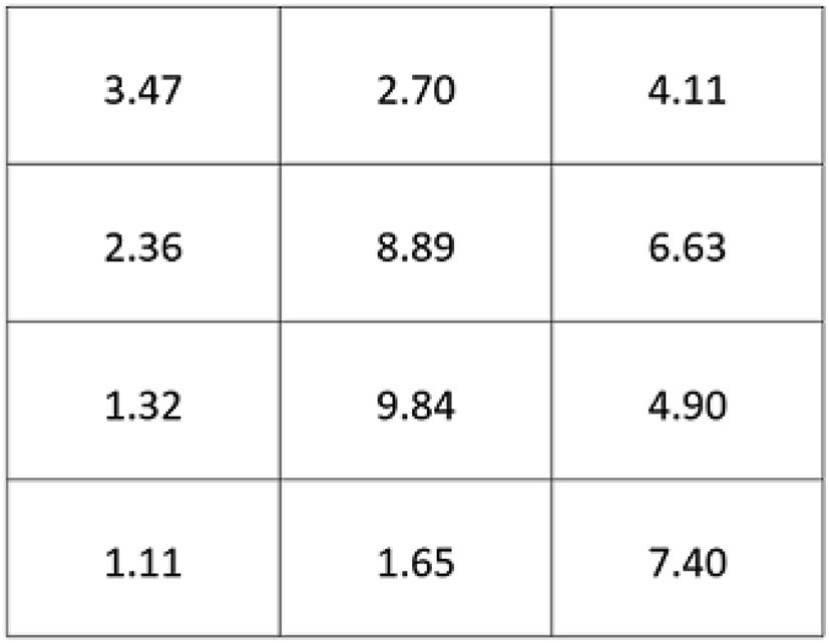
Example of a matrix with 12 three-digit numbers. Respondents were instructed to detect the two numbers that summed up to 10 (i.e., 8.89 and 1.11).

We created a PC between the experimenter and the participant by first establishing the experimenter’s obligation (i.e., provide payment in return for performance) and the participant’s obligation (i.e., solve mathematical tasks). Given that a PC is defined as a cognitive schema containing an individual’s perceptions of their obligations and those of another party^3^.we used this explanation as the core of the PC between the experimenter and the participant. Second, participants were asked to complete a battery of questionnaires to assess recent alcohol or drug consumption or any medical condition that may have an impact on physiological data (e.g., a heart condition). Third, we instructed participants to perform a computer task programmed in E-prime, a software program used for designing and running psychological experiments^62^. The participants were told that their responses would be monitored by an experimenter located in another room. The computer task consisted of a matrix task (32 matrices in total). Each matrix comprised a set of 12 three-digit numbers, in which respondents were instructed to detect the two numbers that summed up to 10 (see example in Fig. 1).

Participants were told that the experimenter would award them tokens for each completed task based on both the accuracy and the speed of their responses compared to a fictitious norm group. After the completion of each task, a message appeared on the screen displaying the amount of tokens study participants were allocated by the experimenter. What the participants did not know was that the number of tokens was randomly determined in advance by the experimenters and was not based on their actual performance. In all five conditions, each participant received the same number of tokens. Participants were promised to be paid for their performance at the end of the experiment. It was explained that each token was worth 0.10€ and that their pay would depend on the total amount of collected tokens, without informing them of the maximum number of tokens that could be collected.

Through this experimental design, we aimed to reproduce real-work experiences since employees can receive a promotion, a pay raise, or a bonus depending on their performance, which they use to evaluate the fulfillment or breach of their PC. Further, in line with previous experimental research^63,64^, we used payment as a general resource type that satisfies other needs.

Based on a between-person design, participants were randomly assigned in one of five conditions based on the timing of the social account delivery after experiencing a breach: prompt (directly after the breach, *n* = 19), delayed (after one additional block, *n* = 18), late (after two additional blocks, *n* = 18), no social account (control condition 1, *n* = 16), and no PCB induction (control condition 2, *n* = 19). During the entire experiment, participants were connected to a NeXus 10-MKII recording device and pre-gelled Ag/AgCl electrodes to assess HR. As a cover story, we told participants that we were studying the effects of emotions on problem-solving skills.

To prepare for the experiment and to accustom participants to the unfamiliar setting, participants were allowed to complete four practice trials. During this time, participants could ask for additional clarification. The experiment comprised seven experimental blocks in total, each consisting of four matrices. After each block (appr. 10 min), the experimenter communicated the total number of tokens and the corresponding amount of money earned by the participant. The default in these blocks was that the experimenter delivered inducements (i.e., tokens) matching the obligations that were established at the start of the experiment, hence fulfilling the PC. However, a PCB was induced by the experimenter halfway through the experiment (i.e., after four experimental blocks) by announcing that participants would no longer be paid for their performance but they were still expected to complete the experiment until the last exercise block. The three experimental conditions differed from each other in the timing in which participants received a social account following the breach experience. The explanation for the breach that served as a social account remained unchanged throughout the three experimental conditions. Namely, participants in the prompt, delayed, and late social account delivery conditions were told that the experimenter was not able to pay them because he invited too many participants and does not have enough money to pay them all*.* Additionally, there were two control conditions in which no social account was given for the induced breach (control condition 1) or no breach was induced (i.e., obligations were fulfilled in all blocks; control condition 2). Finally, participants’ emotions were assessed through a post-experiment questionnaire, after which they were detached from the sensors and debriefed. At the end of the debriefing, each participant received 10€ in return for their participation (appr. 1.5 h).

### Participants

Ninety first-year undergraduate psychology students participated in this experiment in return for course credit. Most of the participants were female (83.3%) and the average age of participants was 19.77 years (*SD* = 3.85). The majority of the participants had attained a secondary school degree (86.7%), followed by bachelor’s (7.8%) and master’s (5.6%) degrees. A minority of participants were currently employed (15.6%) in addition to their studies.

### Measures

#### Psychological contract breach

We measured perceptions of a PCB in the post-experiment survey using a two-item scale from the Psychological Contract Inventory^65^: “In general, the experimental leader fulfilled their obligations to me.” and “In general, the experimental leader lived up to their promises to me.” Participants rated each item on a 5-point Likert scale, ranging from *Totally disagree* (1) to *Totally agree* (5). The Spearman-Brown correlation between both items was 0.82^66^.

#### Feelings of violation

We measured feelings of violation in the post-experiment survey with Morrison and Robinson’s^7^ four-item scale. An example item is “I feel betrayed by the experimental leader.” Participants were asked to rate each item on a 5-point Likert scale, ranging from *Totally disagree* (1) to *Totally agree* (5). The omega (ω) reliability estimate was 0.90 (CI_95%_ = [0.85; 0.95]).

#### Psychological stress

We used the anxiety—comfort and the depression—enthusiasm subscales from Warr’s^67^ measure of affective well-being. This measure is based on the affective circumplex, which allowed us to assess arousal as a subjective, short-term stress response. High scores on the anxiety-comfort scale reflect negative valence—high arousal affect, whereas high scores on the depression-enthusiasm scale reflect negative valence—low arousal. High scores on both scales have been shown to correlate significantly with general distress^67^. We assessed psychological stress both in the pre- and the post-experiment surveys. Participants were asked to rate the extent to which they currently experienced six emotions for each subscale on a 5-point Likert scale ranging from *Did not experience* (1) to *Experienced strongly* (5). The omega reliability estimates were 0.87 (anxiety-comfort; CI_95%_ = [0.82; 0.92]) and 0.78 (depression-enthusiasm; CI_95%_ = [0.68; 0.88]) in the pre-experiment survey and 0.75 (anxiety-comfort; CI_95%_ = [0.66; 0.84]) and 0.69 (depression-enthusiasm; CI_95%_ = [0.50; 0.88]) in the post-experiment survey.

#### Physiological indicator of stress

Physiological stress was operationalized through HR^68–70^. HR is commonly used as a physiological indicator of stress (e.g.,^71–73^) because exposure to psychological stressors activates an adrenomedullary response that is “characterized by release into the bloodstream of epinephrine and norepinephrine and increases in peripheral responses such as heart rate and blood pressure” (^74^, p. 956). HR was continuously assessed throughout the experiment at 32 samples per second. Before running analyses, we standardized HR scores into *z*-scores and removed outliers with a winsorizing procedure (i.e., the 5% smallest and 5% highest HR values for each individual were replaced by the 5th and the 95th quantile, respectively).

#### Resolution velocity

We assessed subjective perceptions of the timeliness of social account delivery with a single item during the post-experiment survey (i.e., “How did you experience the timeliness of the offered explanation?”). Ratings for this item were − 3 (explanation was offered too soon), 0 (explanation was offered on time), and + 3 (explanation was offered too late). This measure formed a subjective counterpart to our objective measure of social account timeliness, which was based on clock time and the duration between a PCB and the social account.

### Analysis

To analyze the HR data, we used discontinuous random coefficient modeling^75^. This technique is ideal for nested data (observations nested within participants) and when a clear transition or discontinuity exists due to, for example, an experimental manipulation. The technique uses a multilevel regression approach with specifically coded independent variables to model (a) the effects of change over time, (b) the transition, and (c) recovery following the transition on the dependent variable. For this study, we first calculated the average HR scores for each trial. By doing so, we removed differences between participants in response times and ensured that the aggregated HR scores satisfied the equidistance assumption of the discontinuous random coefficient modeling technique. Next, we created time, breach transition, social account transition, breach recovery, and social account recovery variables (see Table 1; see Appendix 1 for more information). We then estimated discontinuous random coefficient models in a Bayesian multilevel framework using the brms package in R^76^. A Bayesian approach to this analysis offers several advantages over a frequentist approach^77^. Importantly, it can handle complex models like ours without running into convergence issues, and it can deal with unbalanced and small sample sizes. We estimated a series of Bayesian multilevel models, starting with an empty model without any independent variables, followed by a model with independent variables (i.e., time, transition, recovery, and condition), a model in which we added random slopes for these independent variables, and finally a model in which we included interaction effects between the condition variable and the other independent variables (i.e., transition and condition). Bayesian multilevel models were estimated with two chains and 3000 iterations (1000 warmup iterations). Rhat values were inspected to assess model convergence(Rhat values are a convergence diagnostic for Markov Chains. If the between- and within-chain estimates do not agree, Rhat values are larger than 1, indicating a lack of convergence. Ideally, Rhat values should be less than 1.05). Importantly, a Bayesian approach does not yield p-values but instead relies on 95% credibility intervals. For ease of interpretation, we will use the term *significant* to indicate that a 95% CI does not include zero.

**Table 1:** Coding of time, transition, and recovery variables for discontinuous random coefficient models.

Description	Time	Transition breach	Transition social account	Recovery breach	Recovery social account
Trial 1	0	0	0	0	0
Trial 2	1	0	0	0	0
Trial 3	2	0	0	0	0
Trial 4	3	0	0	0	0
Feedback 1	4	0	0	0	0
Trial 5	5	0	0	0	0
Trial 6	6	0	0	0	0
Trial 7	7	0	0	0	0
Trial 8	8	0	0	0	0
Feedback 2	9	0	0	0	0
Trial 9	10	0	0	0	0
Trial 10	11	0	0	0	0
Trial 11	12	0	0	0	0
Trial 12	13	0	0	0	0
Feedback 3	14	1	1	0	0
Trial 13	15	1	1	1	1
Trial 14	16	1	1	2	2
Trial 15	17	1	1	3	3
Trial 16	18	1	1	4	4
Feedback 4	19	0	1	0	5
Trial 17	20	0	1	0	6
Trial 18	21	0	1	0	7
Trial 19	22	0	1	0	8
Trial 20	23	0	1	0	9
Feedback 5	24	0	1	0	10
Trial 21	25	0	1	0	11
Trial 22	26	0	1	0	12
Trial 23	27	0	1	0	13
Trial 24	28	0	1	0	14
Feedback 6	29	0	0	0	0

Feedback 3 = Breach manipulation + Prompt social account manipulation, Feedback 4 = Delayed social account manipulation, Feedback 5 = Late social account manipulation.

To analyze differences between conditions on the subjective well-being variables and run manipulation checks, we used (mixed) ANOVA. These analyses were performed in R using the afex package^78^. In case ANOVA assumptions were violated, nonparametric tests were used to double-check the robustness of results. Bootstrapped multilevel models were used when assumptions of mixed two-way ANOVA were violated.

All data and analysis scripts can be downloaded from https://osf.io/5p3eq/?view_only=64081842dde543269f5512be66aa37af.

### Ethics approval

This study was approved by the Human Sciences Ethics Committee (ECHW2015-16) of the first author’s university. All methods were performed in accordance with the relevant guidelines and regulations. All participants signed a written informed consent prior to participation.

## Results

### Manipulation checks

We examined if our manipulation of a PCB was successful by comparing scores on measures of PCB perceptions and feelings of violation between the conditions in which a breach was induced (prompt, delayed, late, and no social accounts) and the fulfillment condition. A one-way ANOVA confirmed significant differences existed in breach perceptions between the conditions in the experiment (*F*(4, 85) = 4.25, *p* < 0.001, *η*^*2*^_*G*_ = 0.17, *η*^2^_*G*_CI_95%_ = [0.02, 0.28]) (Since the homogeneity of variances and normality assumptions were violated, we used a nonparametric test to check if these results were robust. A Kruskal–Wallis rank sum test confirmed that significant differences existed between the conditions (χ^2^(4) = 15.32, *p* = 0.004)). The general eta-squared (*η2G*) is recommended as an effect size measure for repeated measurement designs, with values of 0.02 indicating small, 0.13 medium, and 0.26 large effects (cf.,^79^). We performed two planned contrasts to check if these differences were in the expected direction using a holm correction. The first contrast confirmed that conditions in which a breach was induced scored higher on breach perceptions than the fulfillment condition (*estimate* = − 4.18, *t*(85) = − 4.09, *p* < 0.001). The second contrast compared conditions in which a breach was induced and a social account was offered to the condition in which a breach was induced but no social account was offered but found no significant difference (*estimate* = − 0.16, *t*(85) = −0.19, *p* = 0.85). A second one-way ANOVA confirmed that significant differences in feelings of violation existed between the conditions in the experiment (*F*(4, 85) = 3.11, *p* = 0.02, *η*^*2*^_*G*_ = 0.13, *η*^2^_*G*_CI_95%_ = [0.003, 0.23]) (Since the homogeneity of variances and normality assumptions were violated, we used a nonparametric test to check if these results were robust. A Kruskal–Wallis rank sum test confirmed that significant differences existed between the conditions (χ^2^(4) = 13.32, *p* = 0.01)). We ran the same two contrasts as with the breach perceptions analysis, which confirmed that conditions in which a breach was induced scored higher on violation feelings than the fulfillment condition (*estimate* = − 2.35, *t*(85) = − 3.20, *p* = 0.004), while no significant difference was found between breach conditions with a social account versus no social account (*estimate* = −0.11, *t*(85) = -0.18, *p* = 0.85). Overall, these results confirm that our breach manipulation was successful, while the social account timing manipulation did not influence breach perceptions and feelings of violation.

### Effects of psychological contract breach

Hypotheses 1a and 1b proposed that a PCB relates to increased psychological stress and increased physiological reactivity.

#### Psychological stress

To examine the effects of a PCB on psychological stress, we used two independent t-tests to compare the conditions in which we induced a breach in the fulfillment condition. As dependent variables, we used change scores of anxiety and depression (posttest–pretest). To improve power, we used one-sided t-tests, predicting that the increase in anxiety and depression feelings would be greater in the breach condition compared to the fulfillment condition. Results showed no significant differences between the breach and fulfillment conditions on anxiety (*t*(29.36) = 0.84, *p* = 0.20, *M*_*breach*_ = 0.59, *M*_*fulfilment*_ = 0.43, Cohen’s *D* = 0.21, CI_95%_ = [− 1.91, 0.98]) or depression (*t*(25.35) = 1.68, *p* = 0.053, *M*_*breach*_ = 0.27, *M*_*fulfilment*_ = 0.00, Cohen’s *D* = 0.46, CI_95%_ = [− 1.84, 0.98]). Thus, Hypothesis 1a could not be confirmed.

#### Heart rate

**Figure 2:**
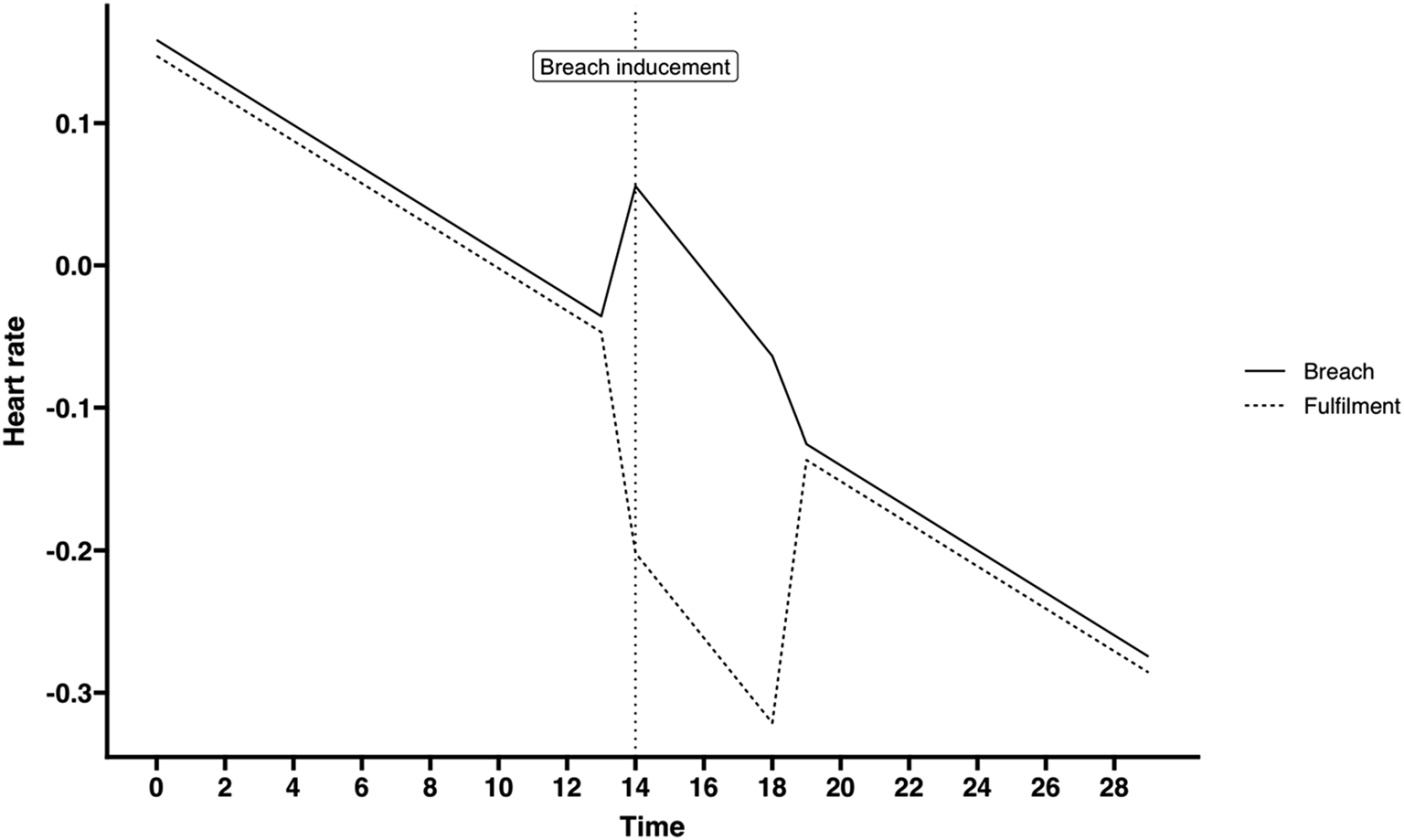
HR trajectories based on Model 1d for breach and fulfillment conditions.

We estimated a sequence (see Table 2) of discontinuous random coefficient models to assess the effect of a PCB—comparing the breach conditions to the fulfillment condition—on HR. We started by estimating an empty model with a random intercept (Model 1a). Next, we estimated a model in which we added the time, transition, recovery, and condition variables as independent variables (Model 1b). Next, we allowed the effect of time, transition, and recovery to vary among participants (Model 1c). In the final model, we added the interaction effect of transition and recovery with condition (Model 1d). We did not include an interaction effect between time and condition, as it was theoretically implausible that condition would affect the HR trajectory prior to the manipulation. The parameter estimates of Model 1d showed a significant interaction effect of transition and condition. To interpret this interaction effect, we used the parameter estimates from Model 1d to plot HR trajectories (Fig. 2). This showed a significantly higher HR in the breach conditions compared to the fulfillment condition in the block following the breach manipulation, thereby confirming Hypothesis 1b.

**Table 2:** Parameter estimates for discontinuous random coefficient models used to estimate effect of breach on HR.

Predictors	Heart rate (model 1a)		Heart rate (model 1b)		Heart rate (model 1c)		Heart rate (model 1d)	
	Estimates	CI (95%)	Estimates	CI (95%)	Estimates	CI (95%)	Estimates	CI (95%)
Intercept	**− 0.05**	**− 0.09 to − 0.02**	**0.16**	**0.12–0.21**	**0.16**	**0.10–0.22**	**0.16**	**0.10–0.22**
Time			**− 0.01**	**− 0.02 to − 0.01**	**− 0.01**	**− 0.02 to − 0.01**	**− 0.01**	**− 0.02 to − 0.01**
Transition			**0.07**	**0.00–0.14**	0.07	− 0.00–0.14	**0.12**	**0.05–0.20**
Recovery			− 0.01	− 0.04–0.02	− 0.01	− 0.03–0.02	− 0.02	− 0.05–0.01
Condition [fulfillment]			− 0.04	− 0.13–0.05	− 0.00	− 0.09–0.09	− 0.01	− 0.09–0.07
Transition × condition							**− 0.24**	**− 0.41 to − 0.09**
Recovery × condition							0.04	− 0.02–0.10
**Random effects**								
σ^2^	0.17		0.15		0.14		0.14	
τ_00_	0.02_person_		0.02_person_		0.07_person_		0.07_person_	
τ_11_					0.00_person.time_ 0.03_person.discontinuity_ 0.00_person.recovery_		0.02_person.discontinuity_ 0.00_person.time_	
ρ_01_								
ρ_01_								
ICC	0.12		0.13		0.25		0.24	
N	89_person_		89_person_		89_person_		89_person_	
Observations	2654		2654		2654		2654	
Marginal R^2^/conditional R^2^	0.000/0.112		0.090/0.203		0.089/0.301		0.094/0.301	

One participant was excluded from this analysis because the trial transitions were not recorded correctly in E-prime during the experiment. Condition contains two levels (0 = breach, 1 = fulfillment). Significant estimates are in bold.

### Effects of social account timing

In Hypotheses 2a and 2b, we propose that the longer the objective timespan from a PCB to the offer of a social account, the less successful the psychological and physiological stress resolution process.

#### Psychological stress

We ran a mixed ANOVA to examine the effects of social account timing on psychological stress with social account conditions (none, prompt, delayed, and late) as a between-person factor, affective subdimension (anxiety versus depression) as a within-person factor, and change scores in emotions as the dependent variable. Perceptions of a PCB were used as a control variable to account for the fact that there could be differences between participants in the degree to which they interpreted the events during the experiment as a breach. We found no significant main effect of social account condition (*F*(3, 65) = 2.11, *p* = 0.11, *η*^2^_*G*_ = 0.06, *η*^2^_*G*_CI_95%_ = [0, 0.20]) or the affective subdimension variable (*F*(1, 65) = 0.05, *p* = 0.83, *η*^2^_*G*_ = 0.00, *η*^2^_*G*_CI_95%_ = [0, 0.06]). There was a significant main effective of the breach control variable (*F*(1, 65) = 6.77, *p* = 0.01, *η*^2^_*G*_ = 0.07, *η*^*2*^_*G*_CI_95%_ = [0.005, 0.24]), such that perceptions of breach were positively related to increases in anxiety and depression. No significant interaction effect of social account condition and affective subdimension (*F*(3, 65) = 0.42, *p* = 0.74, *η*^*2*^_*G*_ = 0.00, *η*^2^_*G*_CI_95%_ = [0, 0.08]) or of the perceptions of breach control variable and affective subdimension (*F*(1, 65) = 1.91, *p* = 0.17, *η*^2^_*G*_ = 0.01, *η*^2^_*G*_CI_95%_ = [0, 0.14]) were observed.

#### Heart rate

**Figure 3:**
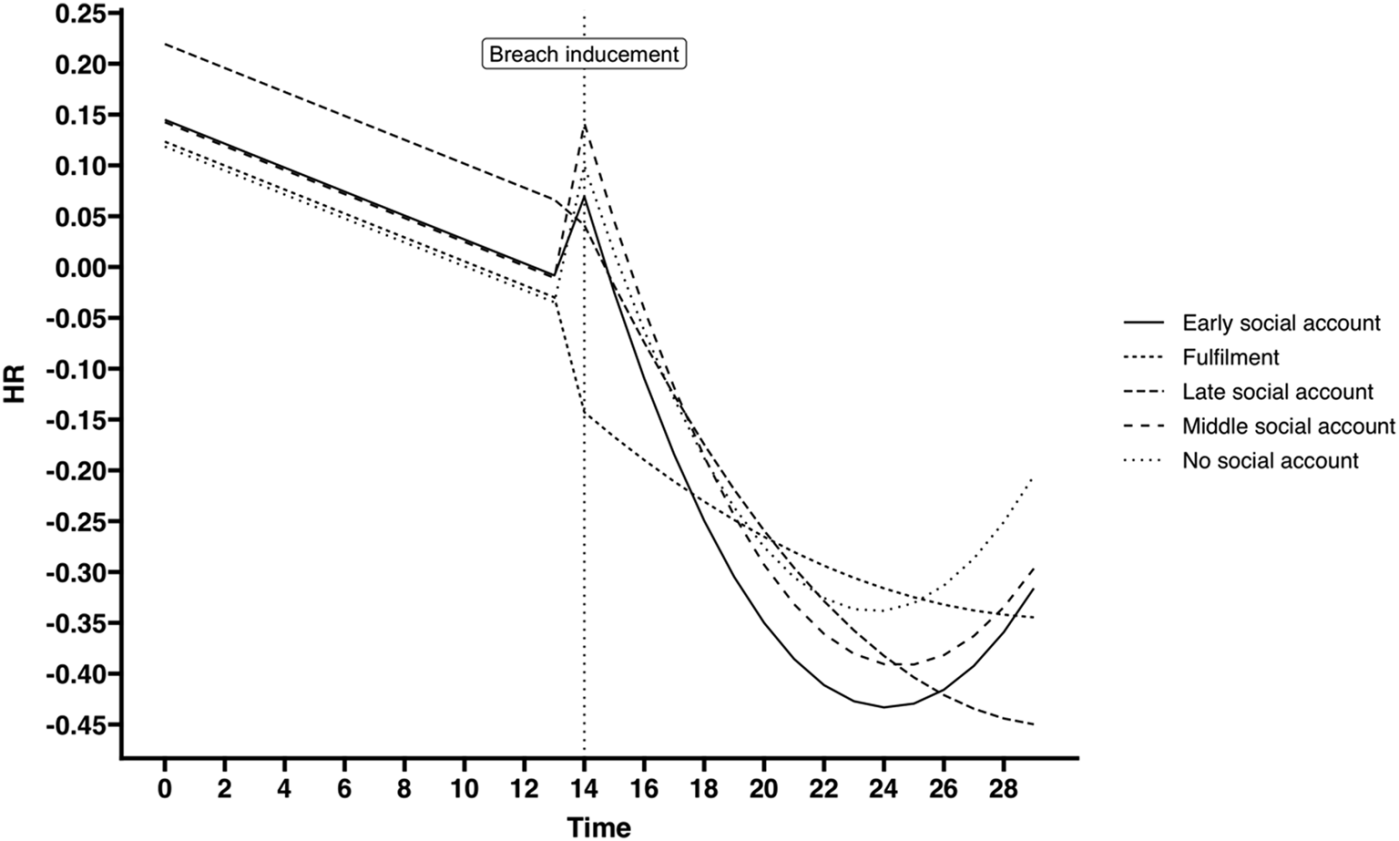
HR trajectories based on Model 2d (Effects of social account timing) for social account conditions.

We estimated a sequence (see Table 3) of discontinuous random coefficient models to assess the effect of social account—comparing the prompt, delayed, late, and no social account conditions to the fulfillment condition—on HR. For this, we used the same approach as with the model to assess the effect of breach on HR, resulting in four models being estimated (Models 2a–2d). Since we examined HR recovery over several blocks following the breach inducement, we also include a quadratic recovery effect to account for nonlinear changes in the trajectory. The parameter estimates of Model 2d show a negative linear trend in HR throughout the entire course of the experiment (i.e., the time parameter). The 95% CI of the transition parameter contains zero, suggesting that this effect is not significant in the fulfillment condition. The interaction effect between the transition parameter and the delayed and no social account conditions shows a significant increase in HR in these conditions when a breach is induced. Next, the recovery and recovery-squared parameters have 95% CIs that include zero, again suggesting that the HR trajectory of participants in the fulfillment condition continued to follow the same linear negative trend as in the first three blocks of the experiment. However, various interaction effects of the recovery and recovery-squared parameters with the condition variables appeared to be significant. In particular, a significant negative linear recovery trend was seen in the prompt, delayed, and no social account conditions. Moreover, a significant quadratic recovery trend was seen in the same conditions. To better understand these trajectories, we used parameter estimates to visualize HR trajectories in each condition. Figure 3 shows that an increase in HR was experienced in all breach conditions, aligning with our findings in Model 1d. Participants in the fulfillment condition experienced a relatively linear decrease in HR in the last three blocks of the experiment. Participants in the prompt, delayed, and no social account conditions experienced an immediate drop in HR followed by an increase in HR toward the end of the experiment. This increase in HR was strongest in the no social account condition. Finally, participants in the late social account condition experienced a drop in HR but did not experience an increase toward the end of the experiment.

**Table 3:** Parameter estimates for discontinuous random coefficient models used to estimate effect of social account timing on HR.

Predictors	Heart rate (model 2a)		Heart rate (model 2b)		Heart rate (model 2c)		Heart rate (model 2d)	
	Estimates	CI (95%)	Estimates	CI (95%)	Estimates	CI (95%)	Estimates	CI (95%)
Intercept	− 0.05	− 0.09 to − 0.02	0.12	0.04–0.21	0.16	0.07–0.25	0.12	0.01–0.23
Time			− 0.01	− 0.02 to − 0.01	− 0.01	− 0.02 to − 0.00	− 0.01	− 0.02 to − 0.00
Transition			0.06	− 0.01–0.14	0.06	− 0.01–0.14	− 0.09	− 0.24–0.06
Recovery			− 0.05	− 0.07 to − 0.03	− 0.05	− 0.07 to − 0.03	− 0.00	− 0.04–0.03
Recovery^2^			0.00	0.00–0.00	0.00	0.00–0.00	0.00	− 0.00–0.00
Condition [prompt sa]			0.01	− 0.10–0.13	− 0.03	− 0.13–0.07	0.03	− 0.12–0.18
Condition [delayed sa]			0.06	− 0.06–0.18	− 0.00	− 0.10–0.10	0.10	− 0.05–0.25
Condition [late sa]			0.03	− 0.08–0.15	− 0.02	− 0.12–0.09	0.02	− 0.12–0.16
Condition [no sa]			0.05	− 0.06–0.17	0.01	− 0.09–0.11	− 0.00	− 0.15–0.14
Transition × condition [prompt sa]							0.19	− 0.02–0.41
Transition × condition [late sa]							0.09	− 0.12–0.30
Transition × condition [delayed sa]							0.26	0.07–0.48
Transition × condition [no sa]							0.25	0.04–0.47
Recovery × condition [prompt sa]							− 0.07	− 0.12 to − 0.02
Recovery × condition [late sa]							− 0.04	− 0.09–0.01
Recovery × condition [delayed sa]							− 0.08	− 0.13 to − 0.03
Recovery × condition [no sa]							− 0.07	− 0.12 to − 0.01
Recovery^2^ × condition [prompt sa]							0.00	0.00–0.01
Recovery^2^ × condition [late sa]							0.00	− 0.00–0.00
Recovery^2^ × condition [delayed sa]							0.00	0.00–0.01
Recovery^2^ × condition [no sa]							0.00	0.00–0.01
**Random effects**								
σ^2^	0.17		0.15		0.13		0.13	
τ_00_	0.02_person_		0.02_person_		0.07_person_		0.07_person_	
τ_11_					0.00_person.time_ 0.03_person.discontinuity_ 0.00_person.recovery_		0.00_person.time_ 0.03_person.discontinuity_ 0.00_person.recovery_	
ρ_01_								
ρ_01_								
ICC	0.12		0.14		0.28		0.28	
N	89_person_		89_person_		89_person_		89_person_	
Observations	2654		2654		2654		2654	
Marginal R^2^/conditional R^2^	0.000/0.112		0.106/0.215		0.103/0.333		0.119/0.340	

One participant was excluded from this analysis because the trial transitions were not recorded correctly in E-prime during the experiment. Condition contains five levels (fulfillment, no social account, prompt social account, delayed social account, and late social account), with fulfillment used as comparison group.

### Effects of resolution velocity

Hypotheses 3a and 3b stated that high-resolution velocity will be associated respectively with more successful psychological and physiological stress resolution compared to low-resolution velocity.

#### Psychological stress

We ran a Bayesian multilevel regression model in which we used change scores in anxiety and depression between pretests and posttests as the dependent variable. As independent variables, we included resolution velocity, affective subdimension (anxiety vs. depression), and the interaction effect between both variables. We also included breach perceptions as a control variable. We accounted for the nested structure in the data (i.e., observations nested within individuals) and estimated models with random intercepts. No significant main effect of resolution velocity was found (*estimate* = − 0.0.01, 95% CI = [− 0.14, 0.12]). However, we did find a significant effect of affective subdimension (*estimate* = − 0.29, 95% CI = [− 0.51, − 0.07]), meaning that change scores were larger for anxiety compared to depression. We also found a significant effect of breach perceptions (*estimate* = 0.22, 95% CI = [0.05, 0.38]), such that change scores increased as breach perceptions grew stronger. Importantly, the interaction effect between resolution velocity and affective subdimension was not significant (*estimate* = − 0.05, 95% CI = [− 0.18, 0.09]), lending no support for Hypothesis 3a. As an additional sensitivity check, we reran these analyses using absolute values of resolution velocity. Again, no significant effect of resolution velocity could be observed.

#### Heart rate

We estimated a sequence of discontinuous random coefficient models to assess the effect of resolution velocity on HR. For this, we used the same approach as with the model to assess the effect of a breach on HR and social account timing on HR, resulting in four models being estimated (Models 3a–3d). We, again, include a quadratic recovery effect to account for nonlinear changes in the trajectory. As can be seen in Table 4—Model 2d, none of the interaction effects between resolution velocity and the transition, recovery, or recovery-squared parameters were significant, as all 95% CI included zero. As an additional sensitivity check, we reran this analysis with the absolute values of resolution velocity to account for the notion that participants may find social accounts that are either too slow or too fast undesirable. However, this resulted in similar findings, with none of the interaction effects being significant. Hence, we could not observe a significant impact of resolution velocity on HR trajectories, offering no support for Hypothesis 3b.

**Table 4:** Parameter estimates for discontinuous random coefficient models used to estimate effect of resolution velocity on HR.

Predictors	Heart rate (model 3a)		Heart rate (model 3b)		Heart rate (model 3c)		Heart rate (model 3d)	
	Estimates	CI (95%)	Estimates	CI (95%)	Estimates	CI (95%)	Estimates	CI (95%)
Intercept	− 0.05	− 0.09 to − 0.02	0.15	0.08–0.23	0.15	0.07–0.24	0.14	0.05–0.23
Time			− 0.01	− 0.02 to − 0.00	− 0.01	− 0.02 to − 0.00	− 0.01	− 0.02 to − 0.00
Transition			0.08	− 0.01–0.18	0.09	− 0.02–0.19	0.11	− 0.00–0.22
Recovery			− 0.07	− 0.09 to − 0.04	− 0.07	− 0.09 to − 0.04	− 0.06	− 0.09 to − 0.04
Recovery^2^			0.00	0.00–0.01	0.00	0.00–0.01	0.00	0.00–0.01
Velocity			0.00	− 0.03–0.03	0.00	− 0.03–0.03	0.02	− 0.02–0.06
Transition x Velocity							− 0.02	− 0.08–0.03
Recovery x Velocity							− 0.00	− 0.02–0.01
Recovery^2^ x Velocity							0.00	− 0.00–0.00
**Random effects**								
σ^2^	0.17		0.15		0.13		0.13	
τ_00_	0.02_person_		0.02_person_		0.07_person_		0.07_person_	
τ_11_					0.00_person.time_ 0.03_person.discontinuity_ 0.00_person.recovery_ 0.25		0.00_person.time_ 0.04_person.discontinuity_ 0.00_person.recovery_	
ρ_01_								
ρ_01_								
ICC	0.12		0.13				0.25	
N	89_person_		52_person_		52_person_		52_person_	
Observations	2654		1549		1549		1549	
Marginal R^2^/conditional R^2^	0.000/0.112		0.123/0.229		0.123/0.319		0.128/0.321	

## Discussion

In the present study, we aimed to investigate how stress reactions unfold over time after experiencing a PCB. More specifically, we examined how these trajectories are influenced by different organizational social account timings (i.e., prompt, delayed, and late) and the subjective interpretation of these timings, that is, resolution velocity. Although Hypothesis 1a, stating that perceptions of a PCB increase psychological stress, could not be supported, we found support for Hypothesis 1b, meaning that perceptions of a PCB cause an increase in physiological reactivity (HR) compared to perceptions of PC fulfillment but not in subjective appraisals of psychological stress (arousal). Further, our results indicate that after experiencing an increased stress episode due to the disruptive event (i.e., a PCB), all participants showed decreasing stress levels throughout the experiment. This decreased physiological activity might be because participants adapted to the experimental setting, that is, they might have calmed down as they understood how the experiment works, thus making it less stressful. Interestingly, participants still reported higher anxiety and depression feelings when they experienced a PCB. This dissociation between physiological and psychological stress responses noticed in our data was also found in other studies. For example, Campbell and Ehlert^80^ showed that physiological and self-reported stress responses are not systematically linked: less than 30% of the 49 analyzed Trier Social Stress Tests (i.e., a laboratory procedure used to reliably induce stress in human research participants) showed significant correlations between perceived emotional stress variables and physiological responses measured by HR and saliva cortisol. Further, Berndt and colleagues^81^ found a negative correlation between physiological and subjective stress response: before and during a ballroom dancing competition, older dancers showed larger endocrine stress responses but reported lower psychological stress compared to younger dancers. Concerning our findings, we can argue that since participants had more time to reflect on the PCB process as they reported psychological stress, they might have made particular appraisals that elicited negative emotions. Appraisal theories (e.g.,^82,83^) claim that appraisals precede and elicit emotions. In the case of a PCB, research has demonstrated that when individuals are confronted with challenging or unexpected outcomes, they will engage in an interpretation process in which cognitive appraisals of such events are made (e.g.^7,84,85^,). Therefore, additional research is required to study the dissociation between physiological and psychological stress responses that might emerge due to the cognitive appraisal process participants go through once a PCB has been perceived or due to other factors such as habituation to the experimental setting that diminishes physiological activity over time. Alternatively, we need to acknowledge that the differential effect on HR and subjective appraisals of stress may be due to the experimental design. For example, by measuring psychological stress after the trials during which physiological measures were obtained, participants may have started to relax.

The effect of social account timing could not be confirmed based on the subjective appraisals of psychological stress, offering no support for Hypothesis 2a. However, Hypothesis 2b could be partially supported based on the HR data, as we found a significant difference between conditions in the (nonlinear) recovery trajectory following a PCB. Considering our results of HR, we conclude that offering no social account appears to be worse for stress recovery compared to conditions in which a social account was offered. Interestingly, we observed that in all social account conditions, except for the late social account condition, participants’ HR rebounded, resulting in higher physiological reactivity toward the end of the experiment. Surprisingly, the late social account condition seems prima facie to be the most beneficial in terms of stress recovery (i.e., HR does not rebound) compared to the prompt and delayed social account conditions. However, we argue that this lack of HR increase toward the end of the experiment may be due to the insufficient time we had after the last trial to capture a potential rebound similar to the other conditions. Indeed, the curvilinear recovery trajectories following a social account observed in the prompt and delayed social account conditions might indicate a form of “anticipation of breach.” In other words, the social account may immediately reduce stress when it is offered but the ongoing process of interpretation development might give rise to uncertainties about the unstable dimension of the breach’s cause and, in turn, trigger a delayed physiological reaction. Indeed, previous research has shown that the anticipation of a stressful event can be just as stressful as the event itself^86,87^.

Finally, our study did not demonstrate a significant effect of resolution velocity on stress resolution, offering no support for Hypotheses 3a and 3b. This might be due to the way this variable was operationalized in the current research, that is, by asking participants in the post-experiment questionnaire to subjectively evaluate the timeliness (too early, timely, or too fast) of the provided explanation. This operationalization is based upon Carver and Scheier’s^88^ proposal that velocity is regulated by a separate feedback system (the metasystem), which compares actual against ideal velocity. However, because resolution velocity was measured after the timing manipulation, it may have been influenced by the latter, making it less effective at assessing participants’ subjective perceptions of time. An alternative could be to measure participants’ general preferred rate of discrepancy reduction (i.e., resolution velocity) *prior to* the experiment to avoid interference with the manipulation of timing.

### Theoretical implications

While we have a wealth of knowledge on associations between a PCB, on the one hand, and emotional, attitudinal, and behavioral outcomes, on the other hand, scholars know little about how long those outcomes last, how they interact and influence each other, or in which sequence they unfold. We, therefore, respond to recent calls for more time-sensitive and dynamic research in the PC literature^3,18,20,22^. For instance, this study contributes to the PC literature by introducing a process-oriented approach that complements the current, rather static approach to the causal relationship between a PCB and its consequences. Moreover, by integrating COR theory into PC theory, this research contributes to the empirical testing of the role of resources in the different post-violation outcomes proposed by Tomprou et al.^22^.

Furthermore, by examining stress recovery as a *process*, that is, focusing on both immediate and delayed responses, we further contribute to the stress literature. Indeed, beyond examining short-lived increases in stress experiences, studying how stress unfolds over time allows us to identify different trajectories, such as brief, reoccurring, or prolonged chronic experiences of high stress levels. Discerning these reactions is crucial since (chronic) stress severely impacts individuals’ well-being (e.g., depression, chronic diseases, symptoms of infectious diseases, or allergic reactions^89^).

### Limitations and future directions

Despite the highly controllable environment that a laboratory setting provides us, we encourage future research to replicate our findings in field settings by examining employees in organizations. This will inevitably offer a larger variety of PCB types (i.e., both relational and transactional) and alternative recovery efforts. Moreover, field research allows the capture of broader and more realistic employment exchanges based on multiple contributions and in a more complex environment. As previously mentioned, the PC is a dynamic phenomenon that is continuously adjusted based on prior experiences and the prospect of future ones. Examining a PCB and its unfolding consequences in organizations can be achieved by using experience sampling methodology, which allows repeated measurements over a period of time.

Further, previous theoretical work suggested that the interpretation given to a PCB has an important impact on individuals’ responses to the breach^2,7,90^. Moreover, while we examined the role of social account timing in stress resolution following a PCB, we did not test the mechanisms that potentially explain the impact of social accounts (timing), such as interactional justice, self-worth, and positive and negative affect. We, therefore, encourage future research to integrate an attributional and/or a cognitive appraisal perspective in the study of stress resolution processes and to examine the mechanisms linking social accounts to changes in stress. This could allow us to gain knowledge on how and why social accounts alter individuals’ stress reactions.

Next, the present research focused on the timeliness of social account delivery and not on the different types of social accounts. One may argue that, depending on the type of social account (i.e., mitigating, exonerating, or reframing^91^), its timeliness will have a different impact on stress resolution. We, therefore, encourage future research to explore this avenue.

Moreover, the timeliness of the social account delivery (i.e., prompt, delayed, late) was measured through short time intervals during the experiment, all within 1 h. In a real-work setting, it may take days, weeks, or even months before a social account is offered. As stress may dissipate over time, even in the absence of a social account, the impact of social account timing on stress recovery may change as more time passes. Based on this, we recommend future research to replicate our study in field settings that capture more realistic timespans between perceptions of a PCB and the delivery of social accounts.

Finally, research has demonstrated that not all breaches are salient. That is, the effect of a PCB depends on the type of inducement that is under threat^92^. Individuals who perceive a PCB will inherently go through a cognitive appraisal process through which they evaluate the resources they may have lost. If the breach is evaluated as having poor (personal) implications, the subsequent emotional reaction will be limited^7^. We, therefore, suggest future research to additionally assess which resources are the most valued, and thus, when under threat, will lead to greater and more visible effects. This information will allow us to gain insight into the underlying mechanisms between a PCB and various stress trajectories. Namely, one can argue that depending on the (personal) value accorded to a threatened resource, different recovery processes can be displayed, ranging from highly successful to totally unfavorable resolution processes.

### Practical implications

Our study revealed that a PCB is a cause of stress. In other words, a PCB will not only lead to lower satisfaction and decreased performance, but also influence employee well-being.

Moreover, it appears that offering no response to PCB perceptions leads to delayed stress reactions over time. Therefore, our findings show that addressing a PCB helps to alleviate stress in organizations. Organizations should be aware of the importance of an adequate response to a disruptive event, as stress may not be resolved if no explanation is offered for the disruptive event. This decreased sense of well-being will be harmful to both the employee and the employer.

Further, it takes the active participation of both the organization and the employee to achieve effective stress management^93,94^. We, therefore, highlight the importance of also training employees to be aware of their cognitive appraisals and how they affect subsequent stress reactions. Moreover, employees can be trained on how to use adequate cognitive reappraisal strategies that may protect them against stress and/or help them cope with the stressor using more problem-focused approaches^95,96^.

## Conclusion

Our findings underscore that a PCB can be experienced as a stressful event. Moreover, it seems that explaining why the PCB has occurred will alter the stress resolution process. Moreover, the timing of this social account appears to relate to the stress resolution process, albeit in a more complex way than hypothesized. Overall, leaving individuals with no explanation for the breach will negatively impact their recovery process.

## Supplementary Information

Supplementary Information.
