# Supplementary material for: Examining how different social account timings influence stress resolution in the aftermath of a psychological contract breach
Source: Sci Rep. 2022 Dec 20;12:22021. doi: 10.1038/s41598-022-25728-8 (PMC9768172; doi:10.1038/s41598-022-25728-8)
Supplement: Supplementary file 1 — Supplementary Information. [file 41598_2022_25728_MOESM1_ESM.docx]

**Appendix 1**

A discontinuous random coefficient model explains changes in a variable (Y) over time using three independent variables: a time variable (linear change over time in Y), a transition variable (a sudden shift in Y when an event takes place at a certain point in time), and a recovery variable (linear change over time in Y following an event that takes place at a certain point in time). The event in our experiment is the moment when we induce a psychological contract breach (PCB). To create the time variable, we made an increasing integer variable that starts at 0 for the first trial in the experiment. To create a transition variable, we made a variable that is coded 0 prior to the event taking place and 1 after the event took place. To create a recovery variable, we made a variable that is coded 0 prior to the event taking place and is an increasing integer variable that starts at 0 after the event took place. In our experiment, transition and recovery variables were created to examine the impact of a PCB on the outcome and social account timing on the outcome. For the former, the event is defined as the moment we induced a PCB (during Feedback Moment 3), and we focus on recovery during the block following the breach inducement (ending at Trial 16). For the latter, the event is defined as the moment we induced a PCB (during Feedback Moment 3), and we focus on recovery during the remainder of the experiment (ending at Feedback Moment 6). Table 1 in the manuscript gives an overview of these variables and how they were coded. Importantly, the effects of the transition and the recovery variables on the outcome are relative to the time variable. For example, a nonsignificant recovery variable implies that linear change after the event is not significantly different from linear change prior to the event. For more information on this approach, we refer the reader to Bliese and Lang [75].
